# Role of Endoscopic Ultrasound in Pancreatic Cancer Diagnosis and Management

**DOI:** 10.3390/diagnostics14111156

**Published:** 2024-05-31

**Authors:** Hayley K. Rogers, Shawn L. Shah

**Affiliations:** 1 Division of Digestive and Liver Diseases, University of Texas Southwestern Medical Center, Dallas, TX 75390, USA; 2 Division of Digestive and Liver Diseases, Dallas VA Medical Center, VA North Texas Healthcare System, Dallas, TX 75216, USA

**Keywords:** endoscopic ultrasound, pancreatic ductal adenocarcinoma, endoscopic ultrasound-guided interventions, celiac plexus neurolysis

## Abstract

The emergence of endoscopic ultrasound (EUS) has significantly impacted the diagnosis and management of pancreatic cancer and its associated sequelae. While the definitive role of EUS for pancreatic cancer remains incompletely characterized by currently available guidelines, EUS undoubtedly offers high diagnostic accuracy, the precise staging of pancreatic neoplasms, and the ability to perform therapeutic and palliative interventions. However, current challenges to EUS include limited specialized expertise and variability in operator proficiency. As the technology and techniques continue to evolve and become more refined, EUS is poised to play an increasingly integral role in shaping pancreatic cancer care.

## 1. Introduction

Despite significant advances in imaging technology and precision medicine, pancreatic cancer remains a formidable disease with significant morbidity and mortality. It is among the deadliest cancers in the United States, estimated to compose 8.3% of all cancer-related deaths with a 12.5% five-year survival rate [1]. It is projected that the number of deaths due to pancreatic cancer are set to continue to increase and become the second-highest cause of cancer-related deaths by 2030 [2]. Patients with pancreatic ductal adenocarcinoma (PDAC)—the most common subtype of pancreatic cancer—often present with advanced disease due to the largely asymptomatic nature in the early phase and lack of universal screening modalities in average-risk patients. Approximately 80–85% of patients have unresectable cancer at diagnosis, with symptoms that may include jaundice with or without abdominal pain, unintentional weight loss, and cachexia. With a rise in incidence over the past 20 years, early detection remains crucial for improving patient outcomes, as surgical resection can be curative. Various clinical pathways exist for managing PDAC, ranging from surgical resection with preoperative computed tomography (CT) imaging or magnetic resonance imaging (MRI) with or without biopsy confirmation to neoadjuvant therapy to palliation. Endoscopic ultrasound (EUS) has evolved as an impactful and versatile tool in managing pancreatic cancer and its associated sequelae; however, the optimal utilization of EUS as a diagnostic and therapeutic modality remains unclear per currently available guidelines. Here, we aim to review the role of EUS as a diagnostic, therapeutic, and palliative instrument for pancreatic cancer care.

## 2. Diagnostic Versatility of Endoscopic Ultrasound

Over the past 40 years, EUS has evolved from a diagnostic modality to one with wide-ranging therapeutic offerings [3]. Endoscopic ultrasound remains essential in both the diagnosis and management of pancreatic cancer due to its minimally invasive ability to access the pancreas across the gastrointestinal tract. While advances in CT and MRI technology have increased the identification of pancreatic neoplasms and improved precise characterization, EUS remains a more sensitive imaging modality, particularly in detecting lesions less than 2 cm [4]. Endoscopic ultrasound has been shown to detect preinvasive pancreatic neoplasms and may have a promising role in select patients [5,6,7]. However, at present, only patients with high-risk genetic predisposition or a family history of pancreatic cancer undergo routine surveillance for pancreatic cancer. EUS is the preferred modality for these patients, with the option of using MRI as a noninvasive screening method. In the Cancer of the Pancreas Screening 3 (CAPS3) study, 225 high-risk patients were screened using EUS, MRI, and CT, with 42% having an abnormal study finding. Endoscopic ultrasound was the superior modality of the three, detecting abnormalities in 42% of the patients compared to 33% with MRI and 11% with CT imaging [8].

**Figure 1:**
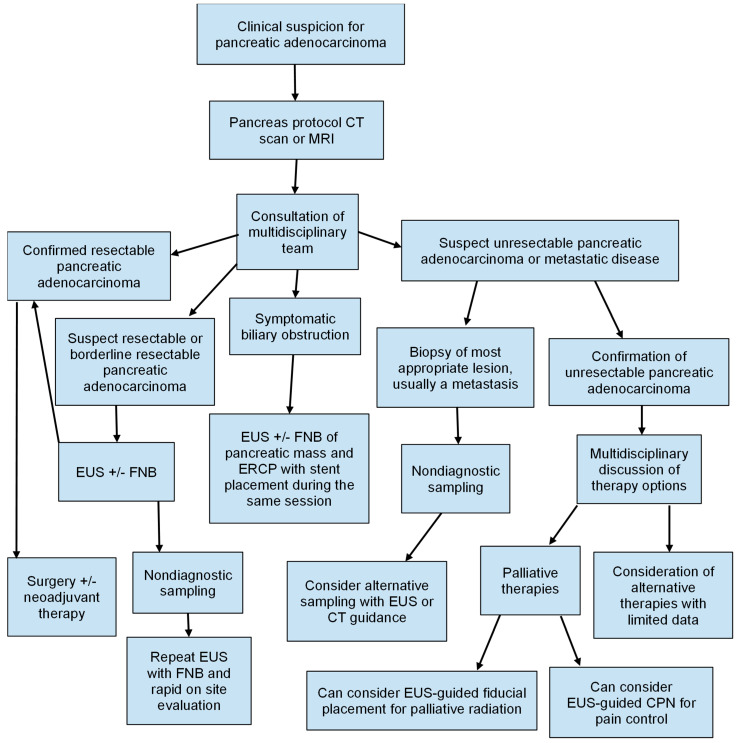
Algorithm for evaluation and management of pancreatic adenocarcinoma adopted from the National Comprehensive Care Network (NCCN) and American Society of Gastrointestinal Endoscopy (ASGE) guidelines. CT—computed tomography: MRI—magnetic resonance imaging; EUS—endoscopic ultrasound; FNB—fine-needle biopsy; ERCP—endoscopic retrograde cholangiopancreatography; CPN—celiac plexus neurolysis.

Upon the suspected diagnosis of a pancreatic neoplasm, tumor, node, and metastasis (TNM), the staging of pancreatic cancer is routinely performed with either a pancreas protocol multidetector CT scan or MRI due to widespread availability. Endoscopic ultrasound can provide medical, radiation, and surgical oncologists with critical information about tumor size, the presence or absence of vascular invasion, and the involvement of nearby structures as a supplementary imaging modality. Dewitt et al. found EUS to be a superior modality in the detection and staging of local, non-metastatic pancreatic cancer (sensitivity for detection 98% with EUS vs. 86% with CT, *p* = 0.012) and similar to CT in a lymph node evaluation [9]. A meta-analysis of 20 studies (*n* = 726 cases of pancreatic cancer) found that the sensitivity and specificity of EUS for the staging of T1 and T2 disease were 0.72 and 0.90, respectively. Additionally, the sensitivity and specificity for staging T3 and T4 disease were 0.90 and 0.72, respectively. While less sensitive and specific for lymph node staging (0.62 and 0.74, respectively), EUS performed better in detecting vascular invasion (0.87 and 0.92, respectively) [10]. Furthermore, the ability to directly sample the pancreatic mass, adjacent and distant lymph nodes, and metastatic foci through EUS in the same session can aid in determining accurate diagnoses and guiding therapy decisions. However, EUS remains largely operator-dependent in terms of its diagnostic imaging interpretation, leading to the review of CT imaging or MRI still being essential at multidisciplinary tumor board meetings. Despite the abovementioned, the pancreas protocol CT scan or MRI remains the recommended staging modality by the National Comprehensive Cancer Network (NCCN) clinical practice guidelines, further stating that EUS can serve as a complementary modality, particularly in cases where vascular invasion or the involvement of nearby structures is uncertain [11] (Figure 1). EUS for the staging of pancreatic neoplasms is likely to continue to evolve as image technology and operator variability improve.

Adjunct features of EUS have aided in further image optimization and enhanced its diagnostic abilities. One such feature is contrast-enhanced harmonics, a technique that can enhance the visualization of vasculature and tissue characteristics by administering an intravenous contrast agent in the form of microbubbles. Contrast-enhanced EUS (CE-EUS) can help differentiate between adenocarcinoma and other solid lesions (e.g., potential benign lesions), as well as better visualize lesions that are obscured by Doppler artifacts and guide improved sampling if prior attempts failed [12]. Similar to cross-sectional abdominal imaging, contrast can be useful to help distinguish hypo-enhanced PDAC from other iso- or hyper-enhanced lesions, such as neuroendocrine tumors and metastatic lesions. Furthermore, the contrast burden is lower than CT imaging and is cleared rapidly through the liver and spleen. In a prospective study, CE-EUS increased the sensitivity of fine-needle aspiration (FNA) from 58.8% to 76.5% (*p* = 0.01) and the adequate sampling rate from 68.8% to 84.9% (*p* = 0.003) [13]. A systematic review and meta-analysis found CE-EUS to have a pooled diagnostic sensitivity of 84% (95% CI, 80–88%) and specificity of 78% (95% CI, 70–84%) [14,15,16]. Contrast-enhanced EUS may also help predict the aggressiveness of PDAC if certain perfusion parameters are met (e.g., lower peak enhancement and wash-in area under the curve) [17]. Another complementary feature is the use of elastography during EUS to assess the stiffness of a pancreatic lesion to improve targeting in fine-needle sampling. Strain elastography with EUS measures tissue distortion in which variability of stiffness is represented by different colors, with stiff tissue represented as blue as compared to normal pancreatic parenchyma, which would be green. This technique is highly user-dependent, but has been shown to have a high sensitivity and low specificity (0.98 [95% CI, 0.96–0.99] and 0.63 [95% CI, 0.58–0.69] for qualitative elastography, respectively) in differentiating malignant solid pancreatic neoplasms from benign lesions [18]. Combining CE-EUS and elastography has been shown to give complementary information; however, it does not appear to offer statistically different diagnostic accuracy as compared to the use of either modality independently [16,19]. Lastly, artificial intelligence has begun to be developed for pancreatic cancer detection. Computer-assisted diagnosis (CAD) to increase the detection rate of pancreatic neoplasms has been demonstrated in multiple studies and appears promising [20,21]. In a systematic review and meta-analysis of 10 studies (*n* = 3529 patients, 34,733 training images), the pooled sensitivity and specificity for deep-learning-assisted EUS for accurately diagnosing pancreatic tumors was 93% (95% CI, 87–96%) and 95% (95% CI, 89–98%), respectively [22]. Artificial intelligence technology has the ability to act as a “second set of eyes” for the endosonographer and potentially reduce interobserver variability and standardize interpretation of EUS images. As these technologies continue to be refined, they are set to become increasingly valuable complementary tools for the endosonographer, improving the detection and characterization of pancreatic neoplasms.

## 3. EUS-Guided Fine-Needle Aspiration and Biopsy

In addition to the identification and staging of pancreatic neoplasms, EUS is ideal for sampling abnormalities within the pancreas. In patients with resectable disease, the NCCN clinical practice guidelines on pancreatic adenocarcinoma state that while EUS-guided sampling is preferred to CT-guided, biopsy proof may not be required in certain cases where radiological and clinical suspicion of resectable pancreatic cancer exists [11]. Conversely, the American Society of Gastrointestinal Endoscopy (ASGE) recommends evaluating suspected pancreatic neoplasms with EUS with or without sampling, which should be individualized to patients contingent on subsequent care and obtaining a multidetector pancreas protocol CT scan [23]. Nonetheless, EUS sampling of pancreatic neoplasms remains the preferred method of tissue acquisition with a high sensitivity and specificity (90.8%, CI 89.4–92%, and 96.5%, CI 94.8–97.7%, respectively), better safety, and a significantly lower risk of neoplastic seeding as compared to the percutaneous approach [24,25,26]. The risks of FNA and fine-needle biopsy (FNB) certainly need consideration, with an overall adverse event rate of 0.5–3%, and include bleeding, pancreatitis, perforation, and abdominal pain [23,25]. A variety of techniques (e.g., slow-pull and suction) and needle sizes (from 19 gauge to 25 gauge) exist for performing high-quality FNA and FNB. There does not appear to be any significant difference in terms of performance between the slow-pull capillary and suction techniques, albeit with an improved histological quality of samples with the slow-pull technique [27,28]. Moreover, in a network meta-analysis by Facciorusso and colleagues, the authors found no tissue sampling technique superior for diagnostic accuracy and sample adequacy for solid pancreatic lesions based on needle type or needle gauge [29]. Additionally, while multiple studies have shown that FNA biopsies provide an adequate yield, the NCCN guidelines recommend the use of newer-generation core FNB needles [11,30]. In a systematic review by Hassan and colleagues of nine randomized controlled trials, EUS–FNB was superior to EUS–FNA for diagnosing pancreatic cancer (OR 1.87) [31]. Furthermore, FNB specimens provide adequate core tissue for histologic purposes, facilitating next-generation sequencing and molecular profiling for precision medicine. While a rapid on-site evaluation (ROSE) may provide immediate preliminary data for the endosonographer, studies have demonstrated a high diagnostic accuracy for pancreatic lesions without a ROSE with newer-generation FNB needles [32]. Ultimately, performing high-quality EUS with good sampling technique (i.e., utilizing the fanning technique, avoiding interposed vasculature, etc.) is crucial.

Endoscopic ultrasound also allows for the sampling of nearby lymph nodes and distant metastatic foci, such as lesions in the liver, if in proximity to the gastrointestinal tract. A precise diagnosis of pancreatic cancer remains paramount, as radiologic characterization does not provide a histologic correlation, and suspecting pancreatic carcinoma off of a CT scan or MRI can lead to a potential misdiagnosis. In a single-center study by Haseeb and colleagues looking at 99 patients with a solid pancreatic mass off of cross-sectional imaging, diagnoses other than adenocarcinoma were found in 25% (25/99) of patients [33]. These diagnoses included benign lesions, neuroendocrine tumors, solid pseudopapillary tumors, and pancreatic metastases. Pursuing a biopsy in patients with suspected pancreatic neoplasm should be discussed with patients and in a multidisciplinary fashion, particularly given the significant risks of surgical resection in a patient with a potentially misdiagnosed tumor or benign lesion.

## 4. EUS-Guided Fine-Needle Tattooing

Submucosal tattooing during endoscopic procedures to demarcate an area of suspected or sampled neoplasm has been a common practice for many years. In 2002, the first report utilizing EUS-guided fine-needle tattooing (EUS–FNT) was published with tattoo placement into a pancreatic insulinoma to assist with laparoscopic resection [34]. Since then, a variety of pancreatic masses have been marked with tattoos using EUS–FNT to assist with surgical resection. A tattoo, typically with 1–2 cc of carbon spot or India ink through a 22- or 25-gauge FNA needle, can be placed adjacent to the lesion to assist with resection margins or injected directly into the lesion [35]. Direct injection can also help enlarge the lesion for better visualization and change its color compared to the surrounding pancreatic parenchyma. Albeit with limited clinical experience without large published studies, the procedure appears safe and efficacious in observational studies, allowing surgeons to readily identify small pancreatic lesions intraoperatively [36,37,38,39]. In one study of 16 patients who underwent EUS–FNT, all but one had visible tattoos during surgery, with one adverse event of a contained hematoma at the injection site [40]. Further, no interference with the histologic evaluation of the surgical specimen was encountered. Large prospective studies are needed; ultimately, coordination with the surgical team is vital to utilize this technique for improved surgical outcomes.

## 5. EUS-Guided Fiducial Marker Placement and Antitumor Therapies

Neoadjuvant chemoradiation therapy may be offered to select patients with pancreatic cancer with advanced disease to improve quality of life and potential survival [11]. To facilitate the more precise targeting of the radiation beams during stereotactic body radiation therapy (SBRT), the EUS-guided placement of radiopaque fiducial markers has emerged as a safe and less invasive approach as compared to percutaneous or surgical placement [41,42,43,44]. The placement of at least three fiducial markers directly into the tumor and/or periphery is recommended by the NCCN guidelines [11]. In a meta-analysis by Patel and colleagues of 11 studies (*n* = 820 patients with pancreatic cancer), the successful placement of fiducials was achieved in 96.27% of patients with an adverse event rate of 4.85%, including a rate of fiducial migration of 4.33% [45]. Other potential complications include pancreatitis, bleeding, and cholangitis when prophylactic antibiotics are not given. Traditionally, fiducials were gold cylinders with a more recent evolution to a coil design. There does not appear to be a difference in migration between the two design types, and both are loaded directly into a 22-gauge FNA needle. However, one study found that the traditional markers had better median visibility scores on CT imaging as compared to those with coiled fiducials (2.00 vs. 1.75, *p* = 0.009) [46]. Preloaded needles with fiducials have also become available.

There has been much interest in EUS-guided fine-needle injection (EUS-FNI) and the targeted EUS-guided treatment of pancreatic tumors. In patients with PDAC that are deemed potentially resectable or borderline resectable, studies have shown that proceeding straight to surgical resection can lead to positive surgical margins and persistent systemic disease recurrence [47]. The NCCN guidelines now recommend that these patients undergo neoadjuvant chemotherapy to help downstage disease burden before undergoing surgical resection [11]. There is much potential for novel EUS therapies to assist with this downstaging, which, in turn, may facilitate a decrease in disease burden and improve overall outcomes. Endoscopic ultrasound-guided tumor ablation using radiofrequency ablation (EUS–RFA) has been described as a treatment modality for non-resectable pancreatic cancers, facilitating selective tumor ablation through thermal coagulation with limited injury to the surrounding tissue. The RFA probe can be inserted through the EUS needle and, using ultrasound guidance, can deliver variable energy levels directly to the lesion, triggering an immune response. Several small studies have shown EUS–RFA to be feasible, safe, and with limited side effects [48,49,50,51,52,53]. One prospective, observational study looking at EUS–RFA in patients with unresectable pancreatic cancer (*n* = 22) combined with chemotherapy demonstrated a median overall and progression-free survival of 24.03 months (95% CI 16–35.8) and 16.37 months (95% CI 8.87–19), respectively. Patients in the study underwent repeated EUS–RFA until the tumor burden was decreased (median of five sessions, IQR 2.66–9.65), followed by two days of chemotherapy. There were four patients with EUS–RFA-related adverse events (3.47%), including one patient with peritonitis and three with abdominal pain, all of which were managed conservatively [54]. There is even some suggestion that RFA may activate the systemic immune response via inflammation, though this has not yet been confirmed [55]. Another modality that utilizes an ablative technique via EUS is photodynamic therapy (EUS–PDT). Patients are initially given a photosensitizing agent intravenously (e.g., verteporfin) that accumulates in tumors, followed by the EUS-guided placement of a photoradiation fiber into the tumor via a 19-gauge needle. A diode laser is then activated, and the tumor is illuminated, which induces local tissue coagulative necrosis by generating cytotoxic oxygen species [56]. Several observational studies of EUS–PDT have demonstrated tumor necrosis in the majority of patients (50–62.5%) with no related adverse events in locally advanced pancreatic adenocarcinoma [57,58]. The median overall increase in the percentage of necrosis of the tumor was 18% (*p* = 0.16) in a single center, prospective phase one study in treatment-naive patients with locally advanced pancreatic cancer. Further, in that study of 12 patients, the median progression-free and overall survival was 2.6 months (95% CI 0.7) and 11.5 months (95% CI, 1.1–16.9), respectively. There have also been a few animal and human studies on the use of laser ablation with fine fibers, but this continues to be experimental [59,60]. Due to the small sample size of these studies and lack of randomized and controlled data, the long-term efficacy of these treatment modalities remains unclear and are not currently recommended in the guidelines for the treatment of advanced pancreatic adenocarcinoma.

Chang and colleagues demonstrated the first use of EUS–FNI with a cytoimplant (i.e., allogeneic mixed lymphocyte cultures) into unresectable pancreatic tumors in 2000 [61]. In the small study of eight patients, two had a partial response in tumor size, while one had a minimal response; however, the follow-up study comparing this technique to gemcitabine was terminated early due to inferior efficacy in the EUS–FNI cohort [62]. Other therapeutic agents utilizing EUS–FNI for unresectable pancreatic adenocarcinoma have been explored, including dendritic cell-based immunotherapy, oncolytic viruses, and direct chemotherapy injection [63,64]. However, studies remain largely limited to animal studies and human case series with limited efficacy and safety data. Another potential therapeutic modality that has been explored is brachytherapy. Interstitial brachytherapy is most commonly used in patients with prostate cancer and is traditionally placed via CT imaging or through ultrasound guidance. Endoscopic ultrasound can be used to directly inject radioactive seeds, most commonly iodine-125, through the lumen of a FNA needle into pancreatic tumors for localized targeted radiation [65]. One study showed improved quality of life as measured by improvement in abdominal pain scores, but no survival benefit [66,67]. In the study group’s long-term follow-up (*n* = 100 patients, mean follow-up 7.8 months), there was no statistical difference in the median survival time between patients with early and advanced pancreatic cancer or between patients with or without pre-treatment chemotherapy. Another ongoing trial is evaluating EUS-guided brachytherapy with phosphorus-32 microparticles as the radio-isotope in combination with gemcitabine and nab-paclitaxel [68,69]. While EUS–RFA, EUS–FNI of anti-tumor agents, and EUS-guided radiation therapy remain promising, given the current knowledge gaps that exist for these aforementioned therapies with a lack of long-term efficacy and safety data, they continue to remain investigational.

## 6. EUS-Guided Celiac Plexus Neurolysis

Patients with pancreatic cancer often present with severe abdominal pain that can be challenging to manage despite opiates, and significantly impair quality of life. Endoscopic ultrasound-guided celiac plexus neurolysis (EUS–CPN) offers patients efficacious pain relief and is recommended by the NCCN for the treatment of refractory cancer pain [11]. Endoscopic ultrasound can readily identify the celiac plexus from the proximal gastric body, located between the celiac trunk and aorta. Celiac plexus neurolysis is typically performed with an injection of a combination of dehydrated 98% absolute alcohol, a neurolytic agent, and 0.25% bupivacaine, an analgesic agent. Three predominant techniques have been described for EUS–CPN, including central injection (anterior to the root of the celiac artery), bilateral injection (bilateral injection at the root of the celiac artery), and direct injection into the celiac ganglia [70,71]. In a meta-analysis by Koulouris and colleagues, the overall pain remission response rate was 68% after 2 weeks and 53% after 4 weeks [72]. There was no significant difference in the response rate among the various techniques, including central injection, bilateral injection, and direct injection into the celiac ganglia. The procedure can be repeated in patients who experience a period of pain relief. Diarrhea, the transient worsening of abdominal pain, and transient hypotension were among the most commonly reported adverse events, though there have been cases of more serious consequences, such as perforation [73,74]. While EUS–CPN does improve pain scores in patients with pancreatic cancer and can be used effectively as an adjunct to opiates, it does not impact overall outcomes and survival [75,76,77,78,79]. It is hypothesized that EUS–CPN does not provide long-lasting pain relief due to neural regeneration over time and the extent of the invasion of the celiac ganglia [80]. Kong and colleagues examined the use of EUS–CPN with ethanol with and without the combination of local brachytherapy with iodine-125 radioactive seeds in a randomized control trial in 10 patients with refractory abdominal cancer pain (*n* = 10, 6 with pancreatic cancer). The authors found that patients who received a combination of EUS–CPN with brachytherapy achieved the same analgesic effect in the early phase as EUS–CPN alone and achieved an overall longer analgesic effect as they did not experience significant pain recurrence during the 3 months of follow-up. Endoscopic ultrasound-guided CPN should be considered for patients requiring high doses of opiates with persistent abdominal pain or medication-related adverse events.

## 7. EUS-Guided Biliary Drainage

Pancreatic cancer results in some degree of biliary obstruction in approximately 80% of patients, requiring the consideration of biliary decompression [81]. Per NCCN guidelines, in patients with symptomatic obstructive jaundice when anticipating a delay in surgery or non-surgical candidates, the EUS-guided sampling of the pancreatic mass and endoscopic retrograde cholangiopancreatography (ERCP) for biliary decompression should be performed during the same session [11]. However, preoperative biliary decompression in those with asymptomatic jaundice likely does not improve surgical outcomes in patients undergoing surgery within the subsequent 1–2 weeks [82,83]. In terms of stent type for those undergoing neoadjuvant therapy, a meta-analysis by Lyu and colleagues demonstrated lower rates of reintervention and recurrent biliary obstruction with metal stents as compared to plastic stents, with no difference in postoperative outcomes [84].

However, ERCP can be technically difficult when malignant strictures exist in the duodenum and/or biliary tree due to pancreatic neoplasms. When transpapillary access cannot be achieved, EUS offers the ability to create a transluminal biliary bypass through either a choledochoduodenostomy, through the duodenum into the common bile duct, or a hepaticogastrostomy, through the gastric lumen into the left intrahepatic duct [85,86]. However, due to the concern of complicating future surgery for patients with resectable or borderline resectable pancreatic tumors, percutaneous biliary decompression remains the mainstay of therapy at present. A multicenter, randomized controlled study comparing ERCP to EUS-guided choledochoduodenostomy in patients with pancreatic cancer (including those with borderline resectable disease) demonstrated no significant difference in stent function, adverse events, oncologic outcomes, including pancreaticoduodenectomy outcomes, and quality of life between the two groups [87]. A meta-analysis of EUS-guided biliary drainage studies demonstrated a pooled technical success rate of 91.5% and an adverse event rate of 17.9%, comprising primarily of bile leaks (4.1%), stent migration (3.9%), and infection (3.8%) [88,89,90]. Further data are needed to elucidate optimal procedural choice with standardization, carefully considering the impact on surgical and oncologic outcomes.

## 8. EUS-Guided Gastrojejunostomy

Patients with pancreatic cancer develop gastric outlet obstruction in 10–25% of cases [91]. Traditional therapies for outlet obstruction in patients with pancreatic cancer include enteral stenting and surgical gastrojejunostomy. Akin to the surgical procedure, EUS can be used to create anastomosis, or gastrojejunostomy (EUS–GJ), in patients with unresectable pancreatic cancer between the gastric lumen and a downstream loop of small bowel beyond the point of obstruction. This is typically performed by distending the downstream bowel with copious fluid, followed by the endosonographic visualization of the dilated loop of bowel from the gastric lumen and deploying a lumen-apposing metal stent (LAMS) to create an anastomosis [92]. Clinical success rates between endoscopic and surgical methods are similar (84% for EUS–GJ, 87% for enteral stenting, and 90% for surgical gastrojejunostomy), with significantly fewer complications with the endoscopic modalities (12% for EUS–GJ, 11.66% for enteral stenting, and 41% for surgical gastrojejunostomy) [93,94,95]. A randomized control trial compared enteral stenting to EUS–GJ and found that the EUS cohort had a significantly lower frequency of reinterventions (29% vs. 4%, *p* = 0.0029), improved stent patency (HR 0.13, *p* < 0.0001), and improved patient symptoms (mean gastric outlet obstruction score at 1 month of 2.41 vs. 1.91, *p* = 0.012) [96]. Furthermore, EUS–GJ has been shown to have shorter procedural times, length of hospital stay, and time to oral intake compared to a surgical gastrojejunostomy [97]. However, perforation rates from misdeployed stents have been reported to be as high as 10%, with other notable adverse events including gastrointestinal bleeding and stent occlusion [98]. The NCCN guidelines recommend the consideration of gastrojejunostomy in patients with unresectable pancreatic cancer and a life expectancy greater than three to six months, as it provides a more durable treatment as compared to enteral stenting [11]. However, the guidelines do not discuss the role of EUS–GJ, as further data are needed to standardize the technique and improve safety characteristics.

## 9. Future Directions of EUS

The potential for artificial intelligence and artificial general intelligence to disrupt EUS’s role in pancreatic cancer management is tremendous. As the technology continues to evolve and iterate, this would transform an endoscopist’s ability to perform a “smart” endoscopy when evaluating a pancreatic lesion. There is potential for fusing cross-sectional abdominal imaging and EUS imaging to help real-time stage pancreatic tumors, assist with the accurate distinction of pancreatic tumors versus benign lesions, and precisely locate areas within unresectable tumors to directly inject therapeutic agents. Additionally, it may be used to assist in tissue sampling, limiting needle passes, and facilitating the expeditious performance of next-generation sequencing and molecular profiling to help with diagnostics and prognostication. Large AI-assisted models are also being developed to help identify potential cancer biomarkers, which is a pressing need for the early detection of pancreatic cancer and screening in high-risk individuals [99].

Also, therapeutic uses of EUS are set to continue to expand and evolve, and endoscopic oncologists are predicted to become increasingly paramount to the comprehensive care of patients with pancreatic cancer. The widespread therapy options for patients with unresectable pancreatic tumors are anticipated to range from ablative therapies to injectable oncolytic agents. Further randomized controlled studies are needed to help determine the optimal techniques for EUS-guided therapies such as gastrojejunostomy creation and biliary drainage. As tools and devices continue to develop and improve, this can only enhance an endoscopist’s ability to provide therapies for treatment and palliation to patients with pancreatic cancer, from the management of gastric outlet obstruction to biliary obstruction to refractory pain. Endoscopic ultrasound can undoubtedly play an increasingly indispensable and critical role in diagnosing, staging, and treating pancreatic cancer.

## 10. Conclusions

Endoscopic ultrasound is a versatile tool that is set to continue to be essential in the management of pancreatic cancer care. Despite the lack of consensus on the optimal use of EUS, it remains a sensitive tool to identify small pancreatic lesions and can offer pivotal diagnostic information, provide therapeutic interventions, and aid in the palliation of patients with pancreatic cancer. Its utilization is predicted to continue to expand as the technology, tools, and interobserver variability evolve. Ultimately, given the complex disease process with varying clinical sequelae, a multidisciplinary approach should be taken in patients with pancreatic cancer with a thoughtful consideration of the complementary diagnostic and therapeutic functions of EUS.
